# HTLV-1 infection in solid organ transplant donors and recipients in Spain

**DOI:** 10.1186/s12879-019-4346-z

**Published:** 2019-08-09

**Authors:** Carmen de Mendoza, Lourdes Roc, Rafael Benito, Gabriel Reina, José Manuel Ramos, Cesar Gómez, Antonio Aguilera, Manuel Rodríguez-Iglesias, Juan García-Costa, Miriam Fernández-Alonso, Vicente Soriano, on behalf of the Spanish HTLV Network, C. Rodríguez, M. Vera, J. del Romero, G. Marcaida, M. D. Ocete, E. Caballero, I. Molina, A. Aguilera, J. J. Rodríguez-Calviño, D. Navarro, C. Rivero, M. D. Vilariño, R. Benito, S. Algarate, J. Gil, R. Ortiz de Lejarazu, S. Rojo, J. M. Eirós, A. San Miguel, C. Manzardo, J. M. Miró, J. García, I. Paz, E. Poveda, E. Calderón, D. Escudero, M. Trigo, J. Diz, M. García-Campello, M. Rodríguez Iglesias, A. Hernández Betancor, A. M. Martín, J. M. Ramos, A. Gimeno, F. Gutiérrez, J. C. Rodríguez, V. Sánchez, C. Gómez Hernando, G. Cilla, E. Pérez Trallero, J. López Aldeguer, L. Fernández Pereira, J. Niubó, M. Hernández, A. M. López Lirola, J. L. Gómez Sirvent, L. Force, C. Cifuentes, S. Pérez, L. Morano, C. Raya, A. González Praetorius, J. L. Pérez, M. Peñaranda, S. Hernáez Crespo, J. M. Montejo, L. Roc, A. Martínez Sapiña, I. Viciana, T. Cabezas, A. Lozano, J. M. Fernández, I. García-Bermejo, G. Gaspar, R. García, M. Górgolas, C. Vegas, J. Blas, P. Miralles, M. Valeiro, T. Aldamiz, N. Margall, C. Guardia, E. do Pico, I. Polo, A. Aguinaga, C. Ezpeleta, S. Sauleda, M. Pirón, R. González, L. Barea, A. Jiménez, L. Blanco, A. Suárez, I. Rodríguez Avial, A. Pérez Rivilla, P. Parra, M. Fernández, M. Fernández Alonso, A. Treviño, S. Requena, L. Benítez Gutiérrez, V. Cuervas Mons, C. de Mendoza, P. Barreiro, V. Soriano, O. Corral, F. Gómez-Gallego

**Affiliations:** 1 Hospital Universitario Puerta de Hierro-Majadahonda, Madrid, Spain; 2 Universidad CEU-San Pablo, Madrid, Spain; 3 Hospital Miguel Servet, Zaragoza, Spain; 4 Hospital Lozano Blesa, Zaragoza, Spain; 5 Clínica Universitaria de Navarra, Pamplona, Spain; 6 Hospital General Universitario, Alicante, Spain; 7 Complejo Hospitalario, Toledo, Spain; 8 Hospital Conxo-CHUS, Santiago, Spain; 9 Hospital Puerta del Mar, Cádiz, Spain; 10 Complejo Hospitalario Universitario, Ourense, Spain; 11 UNIR Health Sciences School and Medical Centre, Calle Almansa 101, 28040 Madrid, Spain

**Keywords:** HTLV-1, Transplantation, Screening, Tropical spastic paraparesis, Adult T-cell leukaemia, Immunosuppression

## Abstract

**Background:**

HTLV-1 infection is a neglected disease, despite infecting 10–15 million people worldwide and severe illnesses develop in 10% of carriers lifelong. Acknowledging a greater risk for developing HTLV-1 associated illnesses due to immunosuppression, screening is being widely considered in the transplantation setting. Herein, we report the experience with universal HTLV testing of donors and recipients of solid organ transplants in a survey conducted in Spain.

**Methods:**

All hospitals belonging to the Spanish HTLV network were invited to participate in the study. Briefly, HTLV antibody screening was performed retrospectively in all specimens collected from solid organ donors and recipients attended since the year 2008.

**Results:**

A total of 5751 individuals were tested for HTLV antibodies at 8 sites. Donors represented 2312 (42.2%), of whom 17 (0.3%) were living kidney donors. The remaining 3439 (59.8%) were recipients. Spaniards represented nearly 80%.

Overall, 9 individuals (0.16%) were initially reactive for HTLV antibodies. Six were donors and 3 were recipients. Using confirmatory tests, HTLV-1 could be confirmed in only two donors, one Spaniard and another from Colombia. Both kidneys of the Spaniard were inadvertently transplanted. Subacute myelopathy developed within 1 year in one recipient. The second recipient seroconverted for HTLV-1 but the kidney had to be removed soon due to rejection. Immunosuppression was stopped and 3 years later the patient remains in dialysis but otherwise asymptomatic.

**Conclusion:**

The rate of HTLV-1 is low but not negligible in donors/recipients of solid organ transplants in Spain. Universal HTLV screening should be recommended in all donor and recipients of solid organ transplantation in Spain. Evidence is overwhelming for very high virus transmission and increased risk along with the rapid development of subacute myelopathy.

## Background

HTLV-1 infection is a neglected disease. As with other human retroviruses, i.e. HIV, acute infection is universally followed by long-lasting persistence of the virus. Clinical manifestations develop in roughly 10% of carriers lifelong, typically as subacute myelopathy which is known as tropical spastic paraparesis/HTLV-1-associated myelopathy (TSP/HAM) [1] or a lymphoproliferative disorder named adult T-cell leukaemia/lymphoma (ATLL) [2]. Screening for HTLV antibodies is recommended in blood donors in some countries. Acknowledging a greater risk for developing HTLV-1 associated illnesses due to immunosuppression [3], screening of transplant donors and recipients is widely being encouraged [4].

Spain is leading solid organ transplantation worldwide. With 47 million people, 5261 transplants were performed in 2017 (kidney 3269), liver (1247), lung (363) and heart (304). Overall it represents 20% of transplants within the European Union. Until recently, the Spanish law only recommended HTLV testing for persons with high epidemiological risk of infection [5]. Herein, we report the experience with universal testing of a large population of donors and recipients of solid organ transplants in Spain.

## Method

A nationwide network of hospitals/clinics/labs was set up in Spain in 1989 and a national register of HTLV-1 cases works since then. Currently, more than 50 centres distributed across the Spanish geography belong to this group. Further information on this network has been reported elsewhere [6]. All hospitals were invited to participate in the current study. Briefly, HTLV antibody screening using chemiluminescence and/or ELISA of all solid organ donors and recipients attended since 2008 was retrospectively analyzed.

### Statistical analysis

All numerical variables are reported as absolute values and percentages. Categorical variables were compared using χ2 or Fisher exact tests whereas non-categorical variables were compared using Student *T*-test or Mann-Whitney *U* tests. All analyses were 2-tailed and only *p* values below 0.05 were considered as significant. All statistical analyses were performed using SPSS software version 16.0 (SPSS Inc., Chicago, IL).

## Results

A total of 5751 individuals were tested for HTLV antibodies at 8 sites. Table 1 records the main characteristics of the study population. Donors represented 2312 (42.2%), of whom only 17 (0.3%) were living kidney donors. The remaining 3439 (59.8%) were recipients. Males were predominant (59.7%). The median age was 57-years old. Spaniards represented nearly 80%, being Latin Americans only 65 (1.1%) and Africans 101 (1.8%). Overall, transplant recipients were more frequently male, younger, and native Spaniards than organ donors.

**Table 1:** Main features of the study population

	Total	Donors	Recipients	p
No. (%)	5751	2312 (42.2%)^a^	3439 (59.8%)	–
Male gender (%)	3435 (59.7)	1234 (53.4)	2201 (64)	< 0.001
Median age (IQR, years)	57 (45–66)	59 (45–71)	56 (45–65)	< 0.001
Country of birth (%)				
Spain	4595 (79.9)	1383 (59.8)	3212 (93.4)	< 0.001
Other EU countries	114 (2)	34 (1.5)	80 (2.3)	–
Africa	101 (1.8)	14 (0.6)	87 (2.5)	< 0.05
Latin America	65 (1.1)	25 (1.1)	40 (1.2)	–
Asia	9 (0.2)	4 (0.2)	5 (0.1)	–
Unknown	867 (15.1)	851 (36.8)	15 (0.4)	–

^a^17 (0.3%) donors were living kidney donors

Overall, 9 individuals were initially reactive for HTLV antibodies. Table 2 summarizes the main characteristics of these patients. Six were donors and 3 were recipients. Using confirmatory tests (western blot and PCR), HTLV-1 was found in only two patients. Another three were positive for HTLV-2. Two samples gave indeterminate results and another two were negative.

**Table 2:** Main features of HTLV-seroreactive individuals

Case	Year	Donor or Recipient	Gender	Age range (years)	Country of birth	Confirmatory test
1	2010	donor	female	35–40	Spain	HTLV-2
2	2011	donor	female	80–85	Spain	negative
3	2011	recipient	male	45–50	Spain	indeterminate
4	2011	donor	male	45–50	Spain	HTLV-2
5	2015	donor	male	35–40	Spain	HTLV-1
6	2015	donor	female	45–50	Colombia	HTLV-1
7	2015	recipient	male	45–50	Spain	HTLV-2
8	2016	donor	male	50–55	Spain	negative
9	2017	recipient	female	25–30	Colombia	indeterminate

The two HTLV-1 positive cases were donors. Thus, the overall rate of HTLV-1 among solid transplant donors in our study was 0.09%. The first individual was a 49-year old female, born in Colombia, diagnosed in 2015, at the time she was assessed to be a kidney living donor. Before moving to the surgical procedure, she was discharged as potential donor following knowledge of her HTLV-1 seropositive status.

The second HTLV-1 case was a 38-year old male, born in Spain, that died in 2015. Both kidneys were inadvertently transplanted. TSP/HAM developed within 1 year in one recipient, despite antiretroviral prophylaxis attempted within the first weeks. The second recipient seroconverted for HTLV-1 but the kidney had to be removed soon due to rejection. Immunosuppression was stopped and to date, 3 years later, the patient remains in dialysis but otherwise asymptomatic.

The three HTLV-2 cases were two donors and one recipient. All had been born in Spain. The last patient died before transplantation. The two donors were finally discharged from transplantation because they were also positive for HIV and hepatitis C.

## Discussion

More than 35 years after the discovery of HTLV-1 [7], donor/recipient screening for the virus remains sporadic or non-existent in most countries. In 1993, the American CDC recommended that persons infected with HTLV be counselled “*not to donate blood, semen, body organs or other tissues*” [8]. Nevertheless, in the year 2009 the recommendation for universal HTLV screening in deceased organ donors was dropped in the United States because of the perception of low HTLV-1 prevalence and low positive predictive value of serologic screening tests [9]. Alongside this recommendation, most international transplant society guidelines currently do not provide any advice on HTLV-1 screening and use of donated organs.

High rates of HTLV-1 have been found in specific groups in non-endemic regions of North America [10] and Europe [11], and decades of migration/immigration and tourism/travelling have altered the demographics of many Western countries. For example, the ongoing refugee migration from the Middle East and Africa would rapidly change the prevalence of HTLV-1 in many European countries. For a while this has been the case with Latin Americans in Spain, given the large flux of immigration facilitated by strong cultural and ancestor links [6].

Since the year 1988 there is a national register of HTLV-1 cases in Spain. A total of 369 cases had been reported until December 2018. Most cases are concentrated around the largest urban areas (Madrid and Barcelona) where the greatest immigrant populations are living. However, HTLV-1-infected persons have been identified across the whole Spanish geography. Although many were immigrants from endemic areas, mostly in Latin America, up to 18% were native Spaniards [6].

The rapid development of subacute myelopathy was seen in one of our recipients from a kidney from the HTLV-1 positive cadaveric donor. Similar cases of subacute myelopathy shortly after transplantation have already been reported in the literature [12–14], including a series of recipients from a single donor in Spain [15]. Myelopathy was originally reported in a heart transplant recipient in France following HTLV-1 acquisition from contaminated blood transfusions during the surgery [16]. Transplant immunosuppression seems to play a major role in the accelerated disease progression in this setting [17].

In response to the worldwide evolving foci of HTLV-1 infections and the very poor prognosis of post-transplant virus-associated illnesses, there are urgent calls for a broader HTLV-1 screening of live and -more difficult- deceased organ donors. Organ procurement organizations and transplant programs should determine local prevalence to guide HTLV-1 screening efforts [10]. Targeted screening of potential high-risk living (and deceased) donors for HTLV-1 has been recommended by some authors [13]. Suggestions also have been put forward for national or international registries of all HTLV-1-affected transplants [17]. Alerted to the dangers of rapid-onset TSP following HTLV-1-infected organ transplants, Japan began in 2014 screening for HTLV-1 of all kidney donations. Similarly, the United Kingdom issued new transplantation guidance on HTLV-1 screening of cadaveric solid organs in 2011. Finally, the Global Virus Network has recently called for more systematic HTLV-1 screening before solid organ transplantation everywhere [4].

The screening costs for HTLV-1 are small in comparison with the cost of post-transplant illnesses and/or death associated with TSP or ATL following HTLV-1 infection [18]. For all these reasons, the American and European CDCs along with other health prevention agencies should urgently update their policy recommendations on organ transplant HTLV-1 screening. In the meantime, some transplant centres around the world, including a few in Spain, have already implemented “rapid” (or “urgent”) HTLV-1 testing of all deceased organ donors. In parallel, diagnostic companies should design rapid tools (i.e., *point-of-care* (PIC) assays) and improve the specificity of HTLV-1 screening tests to minimize unwanted organ discharge.

Our findings call into question the current view that anti-HTLV screening of donated organs is not needed or just recommended when there is suspicion, as it is recommended in Spain [5]. This opinion is based on the assumption that HTLV-1-associated diseases will develop only in a small proportion of carriers and that progression to disease is slow compared with the average lifespan of humans and therefore poses no major threats to public health. In the transplant setting, the very high risk of transmission and the high rate along with short-term for developing HTLV-1 disease most likely results from immunosuppressive therapy.

## Conclusion

We report a low but not negligible rate of HTLV-1 infection among donors and recipients of solid organ transplants in Spain. Of note, unaware HTLV-1-infected donors were not exclusively foreigners from highly endemic areas but native Spaniards that most likely have been exposed to HTLV-1 by sexual contact. Therefore, universal HTLV screening should be recommended in all donor and recipients of solid organ transplantation in Spain. Evidence is overwhelming for very high -if not uniform- virus transmission and increased risk as well as rapid disease progression, mostly subacute myelopathy.

## Data Availability

The Spanish HTLV database is a public registry ascribed to a public institution, Puerta de Hierro University Hospital, located in Madrid. The person responsible for granting access is Dr. Carmen de Mendoza, first author of this manuscript and current coordinator of the Spanish HTLV Network.

## Abbreviations

CDC: Center for Diseases Control and Prevention
ELISA: Enzyme-linked immune serum assay
HAM HTLV: Associated myelopathy
HTLV-1: Human T lymphotropic virus type 1
TSP: Tropical spastic paraparesis

## References

[1] Gessain A, Barin F, Vernant J (1985). Antibodies to human T-lymphotropic virus type-I in patients with tropical spastic paraparesis. Lancet.

[2] Yoshida M, Seiki M, Yamaguchi K (1984). Monoclonal integration of HTLV provirus in all primary tumors of adult T-cell leukemia suggests causative role of human T-cell leukemia virus in the disease. Proc Natl Acad Sci U S A.

[3] Taylor GP (2018). Human T-lymphotropic virus type 1 infection and solid organ transplantation. Rev Med Virol.

[4] Gallo R, Willems L, Hasegawa H, global virus Network’s task force on HTLV-1 (2016). Screening transplant donors for HTLV-1 and -2. Blood..

[5] Boletin Oficial del Estado (BOE) 2014, November 5, pp. 90536–90538.

[6] de Mendoza C, Caballero E, Aguilera A (2017). Spanish HTLV network. Human T-lymphotropic virus type 1 infection and disease in Spain. AIDS..

[7] Poiesz B, Ruscetti F, Gazdar A (1980). Detection and isolation of type C retrovirus particles from fresh and cultured lymphocytes of a patient with cutaneous T-cell lymphoma. Proc Natl Acad Sci U S A.

[8] Centers for Disease Control and Prevention and the U.S.P.H.S. Working Group (1993). Guidelines for counseling persons infected with HTLV type I and type II. Ann Intern Med.

[9] Stramer S, Notari E, Zou S (2011). HTLV antibody screening of blood donors: rates of false positive results and evaluation of a potential donor re-entry algorithm. Transfusion.

[10] Chang Y, Kaidarova Z, Hindes (2014). Seroprevalence and demographic determinants of HTLV-1 and 2 infections among first-time blood donors – United States, 2000-2009. J Infect Dis.

[11] Mowbray J, Mawson S, Chawira A (1989). Epidemiology of HTLV-1 infections in a subpopulation of afro-Caribbean origin in England. J Med Virol.

[12] Soyama A, Eguchi S, Takatsuki M (2008). HTLV type 1-associated myelopathy following living donor liver transplantation. Liver Transpl.

[13] Ramanan P, Deziel P, Norby S, Yao J, Garza I, Razonable R (2014). Donor-transmitted HTLV-1-associated myelopathy in a kidney transplant recipient - case report and literature review. Am J Transplant.

[14] Younger D (2015). HTLV-1-associated myelopathy/tropical spastic paraparesis and peripheral neuropathy following live-donor renal transplantation. Muscle Nerve.

[15] Toro C, Rodés B, Poveda E, Soriano V (2003). Rapid development of subacute myelopathy in three organ transplant recipients after transmission of HTLV type I from a single donor. Transplantation.

[16] Gout O, Baulac M, Gessain A (1990). Rapid development of myelopathy after HTLV-I infection acquired by transfusion during cardiac transplantation. N Engl J Med.

[17] Taylor G (2013). Lessons on transplant-acquired HTLV infection. Clin Infect Dis.

[18] Jenks P, Barrett W, Raftery M (1995). Development of HTLV-1-associated ATL during immunosuppressive treatment following renal transplantation. Clin Infect Dis.

